# Structure and dynamics of water confined in cylindrical nanopores with varying hydrophobicity

**DOI:** 10.1098/rsta.2020.0403

**Published:** 2021-10-18

**Authors:** Antonio Tinti, Gaia Camisasca, Alberto Giacomello

**Affiliations:** ^1^ Dipartimento di Ingegneria Meccanica e Aerospaziale, Sapienza Università di Roma, Rome, Italy; ^2^ Dipartimento di Matematica e Fisica, Università Roma Tre, Rome, Italy

**Keywords:** nanopores, water, confinement, hydrophobicity, molecular dynamics

## Abstract

We report a detailed study of the main structural and dynamical features of water confined in model Lennard–Jones nanopores with tunable hydrophobicity and finite length (L=26 Å). The generic model of cylindrical confinement used is able to reproduce the wetting features of a large class of technologically and biologically relevant systems spanning from crystalline nanoporous materials, to mesoporous silica and ion channels. The aim of this work is to discuss the influence of parameters such as wall hydrophobicity, temperature, and pore size on the structural and dynamical features of confined water. Our simulation campaign confirmed the existence of a core domain in which water displays bulk-like structural features even in extreme (R=7.0 Å) confinement, while dynamical properties were shown to depend non-trivially on the size and hydrophobicity of the pores.

This article is part of the theme issue ‘Progress in mesoscale methods for fluid dynamics simulation’.

## 1. Introduction

The present work reports a study of the structural and dynamical properties of water in extreme confinement by means of molecular dynamics (MD) simulations. In particular, we provide in-depth analysis of the properties of the extended single-point charge (SPC/E) model of water [1] when confined in cylindrical Lennard–Jones nanopores of finite length and different radii with tunable hydrophobicity and at various temperatures.

The behaviour of water at interfaces and in confinement, especially hydrophobic ones, has been the object of intense research for numerous and diverse reasons, spanning from fundamental investigation to its several implications in biophysics and nanotechnology. It is worth remarking that water *per se* is perhaps the most surprising liquid, with several known anomalies [2]. On the one hand, confinement has been used as an experimental device for probing elusive properties of supercooled water, avoiding the difficulties of pervasive homogeneous ice nucleation in larger samples [3]. On the other hand, confined water is expected to show a possibly broader range of anomalies as compared to bulk water and, thus, its structural and dynamical properties are objects of fundamental interest. The interest in the properties of confined water is further amplified by the extraordinary phenomenology (e.g. transport properties and phase behaviour) observed in a large class of technologically and biologically relevant systems, including (carbon) nanotubes [4–6], nanoporous materials [7–11] and ion channels [12].

Owing to their importance, the structural and dynamical peculiarities of confined water have been the focus of a number of studies, including water in narrow confinement between planar plates [13–16], macromolecules [17], at hydrophobic interfaces [18–22], and confined in several nanoporous systems, such as different varieties of silica pores [10,23–25] and carbon nanotubes [4]. A common trait of these studies resides in the discussion of the existence of two different water populations corresponding to different locations with respect to the wall. The properties of the two populations are observed to depend on the perturbation induced by the wall, and therefore on the chemistry, hydrophobicity and extent of confinement.

The operational distinction between the two water populations is usually obtained by probing the density of water as a function of the distance from the wall. This observable typically shows few oscillations close to the wall which define the contact layers, before becoming uniform far from the wall or in the inner sections of larger nanopores—allowing one to define core/inner or free water. Both in terms of structure and dynamics, core water is similar to bulk water [23,25–28]. The two–three molecular layers corresponding to the oscillations of water density close to the wall were shown to be very distorted in term of the hydrogen bond networks [14,25,29], structural order [21,23,29] and their dynamics was found to be strongly dependent on the hydrophobicity of the wall. In particular, for hydrophilic nanopores water dynamics at the surface is typically slower as compared to the cases of hydrophobic nanopores [25] and core water [29]. When the wall surface presents chemical groups that can strongly hydrogen bond water, surface water was found to become nearly immobile, while the core water still showed bulk-like dynamics [26]. Such local mobility effects were found, in the case of silica pores, to have experimentally observable consequences on the macroscopic flow [30,31].

On the contrary, water at hydrophobic surfaces reaches diffusive dynamics faster than that of surface water on hydrophilic surfaces [14,25]. As the confinement becomes extreme, core water, as defined by the density criterion, disappears. In the extreme case of a single water line found in tight carbon nanotubes, the difference in the dynamics between the hydrophilic and hydrophobic confinement becomes more and more pronounced as the density of water increases [32]. These dynamical features are related to the peculiar transport properties of nanotubes and in particular with the surprisingly large, radius-dependent, slip properties of carbon nanotubes [6,33].

In atomistic simulations, the long-time dynamics is typically probed in axially unbounded nanopores realized by making use of periodic boundary conditions. Nonetheless, in many applications the finite length of the pores is an important feature. This is the case, for instance, with the synthetic and biological pores used in nanopore sensing, in which the nanopores are individually inserted in a membrane and enable transport between its two sides. Water molecules are only transiently present in the pores and there is a continuous exchange with the reservoirs; for such finite-length pores, it is not clear whether confined water molecules are able to reach a diffusive regime and, consequently, whether it is possible to observe a dependence of the dynamics on the hydrophobicity of the wall.

In this study, we focus on model nanopores inserted into a membrane. The model consists of a cylindrical cavity of finite extent, interacting via van der Waals forces with the surrounding water. The surface of the pore has no charges, nor does it form hydrogen bonds with water; the structure is a generic one. This model nanopore is indeed meant to study the general physical effects of confinement and hydrophobicity on the behaviour of water. In recent years, this model has been instrumental in explaining the physical origin of accelerated drying in hydrophobic porous materials [34,35] and how this process can be facilitated by the presence of dissolved hydrophobic gases [36]. In this context, the study of structural features of water in confinement is particularly interesting in the light of earlier observation that structural features of confined water are related to the stability of the confined liquid phase [16]. More specifically, the present results indicate that, even for very high confinements (pore radius R=7.0 Å), a core of water is preserved that displays some degree of bulk-like local structural features. This surprising behaviour seems connected to the lack of surface charges on the pore walls and hydrogen bonds between water and pore atoms. By contrast, translational dynamical properties have been measured to be more sensitive to the confinement, with different dependencies on the pore size and hydrophobicity observed for diffusion along the pore axis and over the pore section.

## 2. Systems and methods

**Figure 1.**
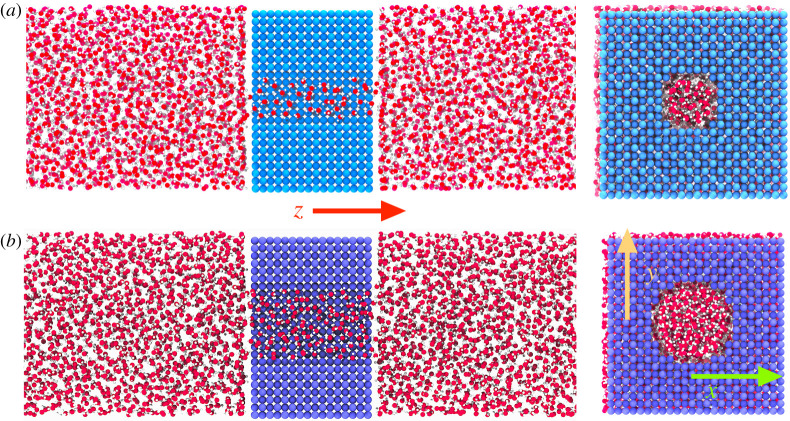
Snapshotsof the molecular dynamics systems employed in the simulations: in light blue a rendering of the smaller pore with radius R=7 Å (*a*), in purple the larger pore with R=10.2 Å pore (*b*). Water molecules are shown in red and white. The temperature is 288.15 K. (Online version in colour.)

MD simulations were employed in order to investigate the structural behaviour of water confined in cylindrical nanopores of length L=26 Å with radii R=7 Å and R=10.2 Å. The pores were obtained by carving a cylindrical hole from a membrane made of a Lennard–Jones crystal (*fcc* symmetry, lattice parameter a=3.5 Å) along the *z*-direction (figure 1). NVT simulations (Nosé–Hoover chains thermostat with a chain length of 3, 1 fs timestep, 100 fs time constant) were performed at four temperatures, T=310.15,288.15,273.15 and 258.15 K. Two water reservoirs, each containing over 5000 water molecules, were placed at both sides of the pore structure, which results in the formation of two planar liquid–vapour interfaces along the *z*-axis (not shown in figure 1); this set-up allowed us to precisely sample two-phase coexistence conditions [37]. Periodic boundary conditions were implemented in the three dimensions.

Water was modelled using the popular extended simple point charge (SPC/E) model [1] and rigidity of the molecules was enforced by using the SHAKE algorithm [38,39]. Water molecules interacted with the pore matrix via Lennard–Jones interactions between the atom of the solid and the oxygen atom of a water molecule: a multiplicative factor *c* was used to modulate the strength of the attractive part of this potential, allowing us to tune the hydrophobicity of the surfaces at will (e.g. [34,40]). Both pore sizes were simulated at three different values of hydrophobicity (c=1.1, c=1.065, c=0.98) which correspond to Young contact angles $\theta_{Y}=85$, 93 and 103°, respectively (the estimation of which was recently discussed in [36]). Structural quantities were obtained by averaging over 2.5 ns of dynamics obtained by discarding the first 0.5 ns from 3 ns trajectories, while dynamical quantities were computed by discarding the first 0.5 ns from longer trajectories, exceeding 14 ns. The present model was conceived in order to offer an adequately simplified, yet sufficiently general model for the study of the fundamental characteristics of water confined within porous materials, particularly those with pores of finite length. This model has been shown to reproduce the wetting features of a wide variety of porous structures, spanning from mesoporous materials [34] to systems of biological interest such as certain ion channels [41] or the solid-state channels used in pore sensing [42].

As is customary for the phase behaviour of water confined in this kind of system, depending on the pore hydrophobicity, one or two (meta)stable states of the system can exist, associated with wet and dry pores. The relative stability is highly dependent on the hydrophobicity, size of the pores and thermodynamic conditions [11]. Initializing the simulation in the wet state is typically sufficient in order to produce consistent sampling of the structure of confined liquid water, without observing evaporation events; yet more hydrophobic pores are characterized by low free-energy barriers for vapour bubble nucleation, making it possible to observe spontaneous vapour nucleation events during the computationally accessible simulation times. Since this is a stochastic process, *a posteriori* some of the considered cases had to be discarded because evaporation occurred.

For the sake of comparison, we simulated a bulk system containing 500 SPC/E water molecules in the NPT ensemble for the same temperatures simulated for the nanopore systems and pressure P=1 bar. The integration step was set to 1 fs. NPT conditions were implemented using the Nosé–Hoover thermostat and the Parrinello-Rahman barostat. Equilibration runs varied from 10 ns at 310.15 K to 30 ns at 258.15 K. NPT runs of 10 ns were used to evaluate properties of bulk water.

## 3. Results and discussions

### (a) Density

**Figure 2.**
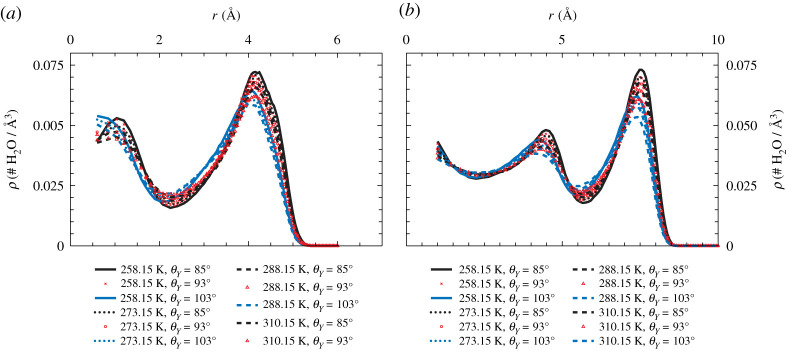
Cylindrical averages of the density of water inside the simulated nanopores as a function of the pore radius, showing the influence on the density of the different confinements, of the wetting characteristics of the pore material and of the temperature. (Online version in colour.)

Water density profiles as a function of the pore radius are reported in figure 2 for the small (panel a) and large (panel b) pores at different degrees of hydrophobicity of the pore walls and at different temperatures, ranging from biological ones (T=310.15 K) down to T=258.15 K. These profiles clearly show the organization of water molecules into circular layers about the pore axis, associated with sharp density peaks. These layers have a thickness of approximately 2.5 Å, thus accommodating one water molecule on average. The number of density layers strongly correlates with the size of the pore: the small pore exhibits a central peak in the density (we will refer to it with the term *inner layer*) and a second peak in contact with the internal wall of the pore (*contact layer*). The larger pore is able to accommodate an additional cylindrical layer of water in between the inner and contact layers, named the *middle layer*. A similar organization into layers is found irrespective of the wetting characteristics of the pore and of the temperature within the ranges of our study. Upon decreasing temperature, water becomes more and more structured with sharper density peaks.

**Figure 3.**
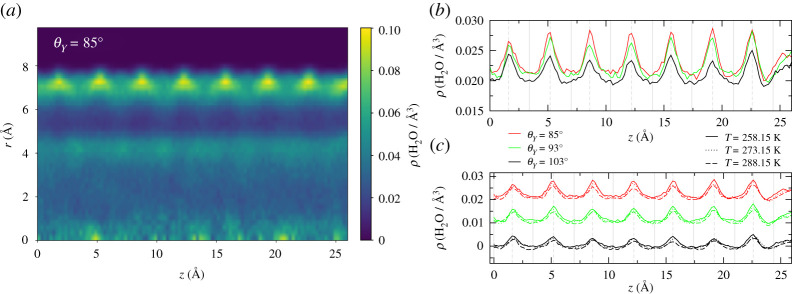
(*a*) Two-dimensional densitymap of water inside the hydrophobic nanopore with R=10.2 Å plotted as a function of the distance *r* along the radius and the coordinate *z* along the pore axis. The wall is located at r=R. (*b*) Density of water in the contact layer along the pore axis for the three hydrophobicities investigated at T=258 K. (*c*) Temperature dependence of the density of water in the contact layer along the pore axis. For clarity, the data for $\theta_{Y}=93^{\circ }$ and 103° have been shifted along the *y*-axis by −0.01 and $-0.02\;{\text{Å}}^{-3}$, respectively. The vertical solid lines in (*b*) and (*c*) mark the position of the pore atoms of the first layer exposed to water, dashed vertical lines the position of the second atomic layer of the pore. (Online version in colour.)

Figure 3*a* shows the two-dimensional density map of water inside the hydrophilic pore at T=258 K, averaged over the azimuthal direction. The middle layer is centred at r∼4.0 Å and the inner (r∼0 Å) water layer of the nanopores does not show a specific pattern along the pore axis; internal layers appear therefore to be homogeneous along the pore length. The contact layer centred at about r∼7.5 Å shows translational order along the *z*-axis parallel to the pore axis displaying a characteristic pattern with eight equally spaced density peaks in contact with the wall. The same pattern is found for the pores with different contact angles, figure 3*b*, but density peaks are less pronounced for more hydrophobic pore walls. This pattern is imprinted by the inner surface of the nanopore, as can be seen from figure 3*b*,*c* which show that contact water density peaks are anti-aligned with the pore atoms belonging to the first layer of the inner pore surface. Upon increasing temperature, the spatial order along the pore axis is preserved; temperature appears therefore not to radically change the structure of the contact layer.

We compute several quantities aimed at characterizing the structure and the hydrogen bonding properties of water. To reflect the strong inhomogeneities in the confined water density, the analysis is done separately in the various layers, defined according to the density minima observed in figure 2: inner and contact and middle layers (the last one only for the larger pores).

### (b) Orientation of water molecules inside the nanopores

**Figure 4.**
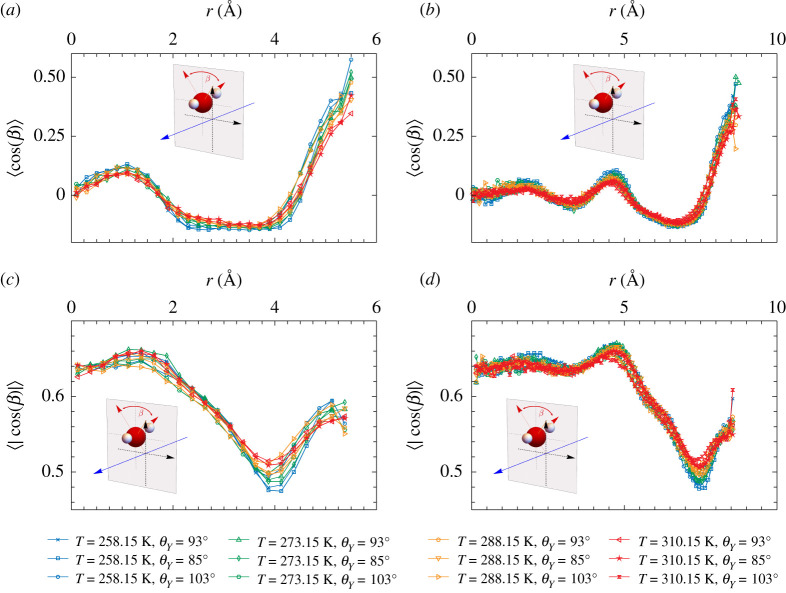
Average water molecule orientations as a function of the radius *r*. The orientation of water molecules is described via the cosine of the angle *β*, as introduced in the main text. (*a*) ⟨cos⁡(β)⟩ as a function of the pore radius *r* for the smaller R=7 Å pore. (*b*) ⟨cos⁡(β)⟩ as a function of the pore radius *r* for the larger R=10.2 Å pore. (*c*) ⟨|cos⁡(β)|⟩ as a function of the pore radius *r* for the smaller R=7 Å pore. (*d*) ⟨|cos⁡(β)|⟩ as a function of the pore radius *r* for the larger R=10.2 Å pore. (Online version in colour.)

We start our analysis of the orientational order of water by probing the local structure of water molecules belonging to the different layers. Figure 4 offers results for the average orientation of water molecules, as a function of the pore radius, hydrophobicity and temperature.

The first orientational parameter we study is the average value of the angle *β* (figure 4). In order to geometrically define the angle *β* for each molecule let us first identify the base plane, i.e. the plane orthogonal to the pore axis passing through the oxygen atom; the angle *β* is now defined, for each water molecule, as the angle between the projection of the H–O–H bisectrix versor on the base plane and the radial vector passing through the O atom.

Results show that the inner portions of the pores display bulk-like behaviour with uniformly distributed values of *β* (i.e. ⟨cos⁡(β)⟩≈0 and $\langle |\cos(\beta )|\rangle \approx t(1/2\pi )\int_{0}^{2\pi }|\cos(\beta )|\text{d}\beta =2/\pi ≃0.6367$). Oscillations are still present and averages slightly deviate from the bulk predictions in correspondence to values of the radii associated to the density minima between the layers. As customary, such peaks are sharper at low temperatures and become more and more smeared as the temperature rises. For both pore sizes, the orientational behaviour manifestly deviates in the outer sections of the pore, and in particular in the contact layer, where the presence of the wall induces a clear ordering. This behaviour corresponds to the rise of cosβ in figure 4 for values of *r* that approach the wall coordinates and was found irrespective of the hydrophobicity of the pore walls.

### (c) Orientational order parameters

The relative spatial arrangement of water molecules is central in the peculiar ability of water to form hydrogen bonds. The different local environments around a water molecule dramatically affect the character of the hydrogen bond networks in the liquid phase. In particular, already at ambient temperature water can be seen as a mixture of two different species fluctuating between two local structures [43]; one species is characterized by high tetrahedral order as the water molecule forms four hydrogen bonds with its four nearest neighbours which leads to a low-density environment. For the other species, instead, the local arrangement around a water molecule is more close-packed and disordered, leading to a high-density local environment [44,45]. These differences in the local environment strongly affect the translational and rotational dynamics of the two species of water [46], whose relative populations are sensitive to the temperature.

**Figure 5.**
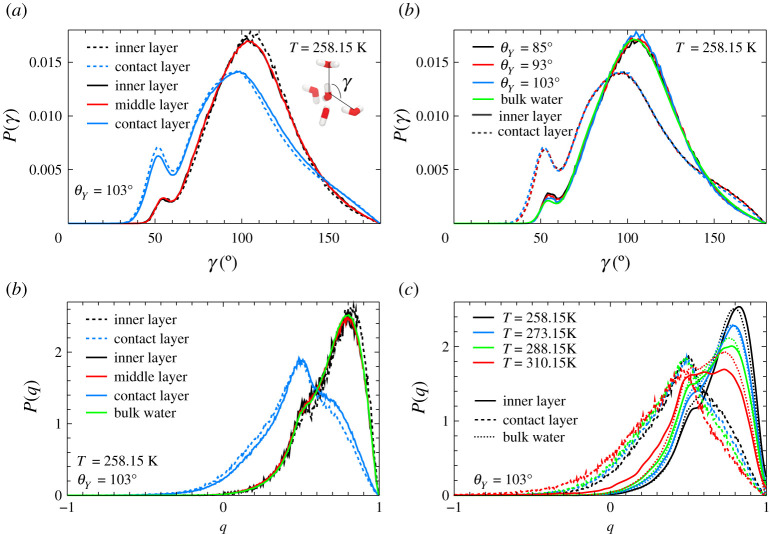
(*a*) Distribution of the *γ* angle between the central molecule and two first nearest neighbours (defined in the pictorial inset) inside the hydrophobic nanopores with R=7 Å (dashed lines) and with R=10.2 Å (solid lines) at T=258.15 K. The position of the central oxygen defines the layer. (*b*) Distribution of the *γ* angle at T=258.15 K for pores with R=7 Å at different degrees of hydrophobicity. (*c*) Distribution of the orientational order parameter *q* of water inside the hydrophobic nanopores with R=7 Å (dashed lines) and with R=10.2 Å (solid lines) at T=258.15 K. (*d*) Temperature behaviour of the *q*-parameter of water inside the hydrophobic nanopore with R=7 Å compared to bulk water behaviour. (Online version in colour.)

For the highly ordered water species an almost perfect tetrahedral arrangement of four water molecules is observed, where the central molecule is surrounded by four nearest neighbour water molecules placed at the corners of a tetrahedron. For a perfect tetrahedron configuration, the angle *γ* between the central molecule and two nearest neighbours is $\gamma =109.5^{\circ }$, see the pictorial inset of figure 5*a*. The angle *γ*, averaged over the first four neighbours, can therefore provide important insights in determining the relative local configurations of water molecules as it can detect strong deviations from the tetrahedral configuration.

In the top left panel of figure 5*a*, we show the distribution of *γ*, P(γ), for a central water molecule belonging to the inner and contact layers of the small hydrophobic nanopore (dashed lines) at T=258.15 K. The two layers are not equivalent in terms of this descriptor. Both distributions show two peaks: the primary peak is located at 106.5° for the inner layer and at 96° for the contact layer. Most of the water molecules in the inner layer are therefore in almost tetrahedral arrangement which favours the formation of hydrogen bonds as in a normal water network. The secondary peak is found at similar positions in the two layers, *ca* 52°–55°; this peak corresponds to neighbour water molecules placed in interstitial positions [23]. By comparing the distributions calculated for the large hydrophobic nanopore (solid lines), we see that the contact layers of the two pores are equivalent. We also found that the middle and inner layers of the large nanopore are equivalent to the inner layer of the smaller pore which indicates that water molecules of the middle layer arrange in a spatially similar manner to the molecules of the inner layer, and that they are both equivalent to the distribution of the inner layer of the smaller pore. At fixed hydrophobicity, therefore, the perturbation induced by the wall extends to the contact layer only. Within the range of our observations, a change in the degree of hydrophobicity of the nanopores does not cause a change in the orientational order among the different layers, see figure 5*b*. In this figure, we also plot P(γ) of bulk water from which we note that the structural properties of bulk water quantified by the angle *γ* are fully recovered by excluding the contact layer. While for the molecules of the contact layer distortions of the hydrogen-bonding network are expected to occur as compared to bulk water because of the missing neighbours on the wall side, it is quite surprising that already from the second molecular layer away from the wall the water molecules organize in such a way that they assume the bulk spatial arrangement.

To quantify the tetrahedral order in one parameter, we computed the orientational order parameter *q*, which is widely used in the water community. *q* is defined using the angle *γ* and an *ad hoc* rescaling [47], as

3.1 $$q=1-\frac{3}{8}\sum_{i=1}^{3}\sum_{j=i+1}^{4}{\left(\cos(\gamma_{ij})+\frac{1}{3}\right)}^{2},$$

and varies between −3 and 1. For an ideal gas, where the neighbours are randomly disposed around the central molecule ⟨q⟩=0, while for a perfect tetrahedron, $\cos(\gamma_{ij})=-1/3$ for all the six angles, leading to ⟨q⟩=1. For this reason, *q* represents a measure of the degree of tetrahedrality.

In figure 5*c*, we show the probability distribution of the *q*-parameter, P(q), for the contact, middle and inner layers in the hydrophobic pores and for bulk water at T=258.15 K. The inner layers of both nanopores and the middle layer of the larger nanopore appear to be equivalent to bulk water. At this low temperature, the distributions for the contact layers and inner/middle layers are bimodal, indicating the presence in each layer of two populations of water molecules with a different orientational order on average. For the contact layer, the distribution is shifted toward lower *q*-values as compared to the inner layer, with a single maximum located at q=0.5 and a shoulder at larger *q*-values. This behaviour closely resembles earlier observations of water in narrow silica nanopores [25]. This reflects the larger population of water molecules having interstitial neighbours ($\gamma \approx 52^{\circ }$–55°, q<0), that we observe in the contact layer as compared to bulk water, see the interstitial peaks in figure 5*b*. Most of the water in the inner/middle layers, instead, is characterized by q=0.8, which corresponds to nearly tetrahedral arrangement [47]. Overall, internal layers appear always more tetrahedrally ordered than contact layers and thus more similar to the bulk structure.

Generally speaking, temperature smears out the tetrahedral order of liquid water. In figure 5*d*, we investigate the orientational order of the water contained in the nanopores upon increasing the temperature. The contact layers (dashed lines) progressively lose the shoulder at larger *q* and become unimodal at the highest temperature. This layer, overall, appears to be weakly dependent on the temperature, presumably because of the small number of water molecules in tetrahedral arrangement which are the most sensitive to temperature. On the contrary, the inner/middle layers shows a larger sensitivity to temperature (solid lines): the population of water molecules at q=0.5 increases progressively upon heating at the expense of highly ordered waters (q=0.8). A similar behaviour is observed in bulk water (dotted lines), but the inner layers of confined water lost tetrahedral order faster than bulk water upon increasing temperature.

### (d) Hydrogen bonding properties

The ability to form hydrogen bonds (HBs) is often thought to be fundamental for the peculiar properties of water [2]. In this work, we adopted a geometrical criterion to define whether an HB exists between two water molecules: the distance between the oxygen atoms of the two candidate molecules is less than or equal to 3.5 Å and the angle formed between the OH bond of the donor water molecule and the vector joining the oxygen atoms of the two candidates is less than or equal to 30°, which guarantees the characteristic high directionality of a hydrogen bond.

**Figure 6.**
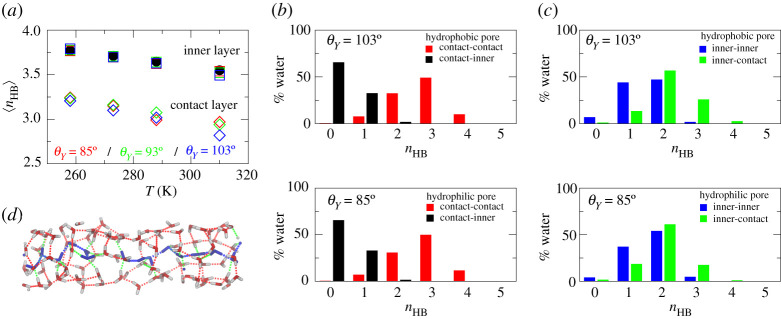
(*a*) Averagenumber of hydrogen bonds (HBs) in bulk water (filled circles), in the inner (empty squares) and contact (empty diamonds) layers of water confined inside the nanopores with R=7 Å as a function of the temperature. Panels (*b*,*c*) show the HB-type analysis performed at T=258 K inside the hydrophobic (top) and hydrophilic (bottom) pore. (*b*) Percentage of contact water molecules engaging *n*_HB_ HBs with another contact (red) and with an inner (black) water molecule. (*c*) Percentage of inner water molecules engaging *n*_HB_ HBs with another inner (blue) and with a contact (green) water molecule. (*d*) Snapshot of the HB network of water inside the hydrophobic small nanopore. Water molecules of the contact layer are shown in red and white and water molecules of the inner layer in blue. HBs (dotted lines) are coloured according to their type: contact–contact (red), contact–inner (green) and inner–inner (blue). (Online version in colour.)

In figure 6, we show the results of the HB analysis performed on water confined inside the smaller pore. We calculated the average number, *n*_HB_, of HBs a single water molecule is involved in for molecules belonging to the inner and contact layers of the pore. In figure 6*a*, *n*_HB_ is plotted as a function of the temperature; the value of bulk water is reported for comparison. For all the simulated hydrophobicities, water molecules of the inner layer form approximately one hydrogen bond more as compared to the water molecules of the contact layer; this value is close to that of bulk water. Upon decreasing temperature, *n*_HB_ increases coherently with what is already mentioned, i.e. that more molecules orientate in bulk-like tetrahedral order.

To further understand the type of the HBs for the contact and inner layer, we reported in figure 6*b* the fraction of water molecules belonging to the contact layer engaging in *n*_HB_ HBs with a molecule belonging to the same layer and with the inner layer. Figure 6*c* shows the analysis of the type of HBs for the inner layer. For the contact layer, around one-third of the molecules forms one HB with molecules of the inner layer. Within the contact layer, instead, water molecules are allowed to take more configurations, with the most frequent case being 3 HBs (half of the population) and the other relevant one 2 HBs; on average, around 2.7 HBs for molecules are formed. Therefore, water molecules belonging to the contact layer are more connected to molecules in the same layer. The inner layer behaves differently, and the two possible types of HB are both relevant for this population of water. The distribution of HBs formed between inner and contact layer molecules shows that such bonds are very important; indeed, the maximum is shifted toward larger values. Almost all the molecules are connected with at least 1 HB with the contact layer. Figure 6*d* shows the hydrogen bond networks of the water molecules inside the small hydrophobic nanopore at T=258 K. The hydrogen bond network between contact water molecules, mainly parallel to the pore wall, is shown in red; HBs connecting contact water molecules to inner water molecules are coloured in green. The network of the inner molecules, in blue, extends along the pore axis displaying a configuration similar to the single-file arrangement observed in narrow carbon nanotubes and biological pores which enable fast proton transfer along the pore [48]. A similar arrangement was observed in more hydrophilic pores.

### (e) Translational dynamics of water

**Figure 7.**
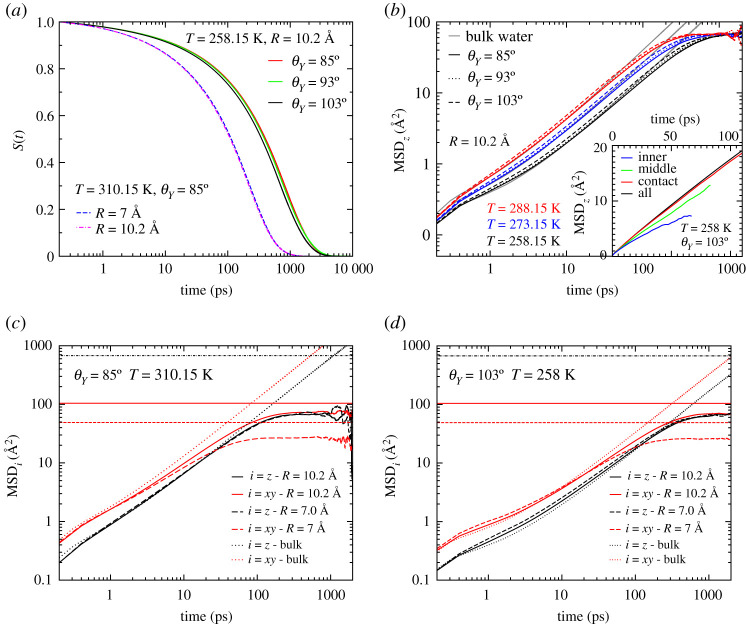
(*a*) Survivaltime correlation function, S(t), of water molecules residing inside the nanopores. (*b*) Axial mean square displacements (${\text{MSD}}_{z}$) of water in the pore with R=10.2 Å at different hydrophobicities and temperatures. In the inset, MSD_z_ calculated separately for the three layers. (*c*,*d*) Effect of the confinement: MSD along the pore axis (*z*) and in the section of the pore (*xy*) for the pores at two different hydrophobicities and temperatures. Horizontal lines in (*c*) and (*d*) mark the maximum allowed distance for *xy* displacements $R^{2}$ (red dashed: R=7 Å, red solid: R=10.2 Å) and *z*-displacements $L^{2}$ (black dot-dashed: L=26 Å). *L* is the length of the pore. (Online version in colour.)

Translational dynamics of water molecules can be probed by computing the mean square displacements, defined as:

3.2 $$\text{MSD}=\left\langle \sum_{i=1}^{N}{\left(r_{i}(t)-r_{i}(0)\right)}^{2}\right\rangle ,$$

where $r_{i}(t)$ is the position of the *i*th oxygen atom of the water molecules at time *t* and *N* is the number of water molecules. In our case, we calculated mean square displacements restricted to the water molecules moving inside the pores. In other words, equation (3.2) is evaluated at time *t* for any water molecules that are continuously present inside the nanopore from time 0 to *t*. Given the finite length of the nanopore, water molecules display a distribution of residence times. We evaluated the survival time correlation function, S(t), by computing the average number of water molecules n(t) that still remain inside the nanopore after a time *t*, S(t)=n(t)/n(0). This quantity is shown in figure 7*a* at T=258.15 K for the three investigated hydrophobicities of the bigger nanopores and at T=310.15 K for the two sizes of hydrophilic nanopores. The relaxation of S(t) provides information about the residence time. These curves show that the residence time depends mostly on the temperature. Hydrophobicity plays a very minor role here, while no dependency on the radius of the nanopore is observed, suggesting that the residence time depends only on the pore length.

Because of the geometry of the confinement, we studied separately the motion in the direction parallel to the pore axis by calculating the axial mean square displacement MSD_z_, and the motion in the pore section normal to the pore axis, by calculating ${\text{MSD}}_{xy}$.

The axial mean square displacements of water molecules MSD_z_ for the pore with R=10.2 Å are shown in figure 7*b* at different temperatures and hydrophobicities. Bulk water results are reported for comparison. At fixed temperature, water moving inside the hydrophobic pore is faster as compared to the other two pores with smaller contact angles. This behaviour is found to be in accordance with recent quasi-elastic neutron scattering experiments performed on porous silica and periodic mesoporous organosilicas [49]. Upon decreasing the temperature, water molecules slow down. Over our range of times and temperatures, confined water shows a temperature-dependent transient regime, 0.2–5 ps, where it moves faster than bulk water. At longer times, bulk water reaches the diffusive regime. The MSD_z_ of confined water, instead, plateaus at a value which corresponds to a linear distance of *ca* 8 Å, imposed by the finite length of the nanopore, which is independent of temperature and hydrophobicity.

Axial diffusion is always dominated by molecules moving in the contact layer; see the inset of figure 7*b* for the hydrophobic pore. This behaviour was also observed for water confined between hydrophobic plates when the density of water is high enough to create three distinct layers [13]. We find this behaviour for all the investigated pores, even for the highest hydrophilicity. In fact, in the case of water confined in hydrophilic confinement, the layers of water closer to the surface were shown to be immobile and even in a glassy state already at high temperature [26,27,49,50]. In silica pores, water of the contact layers can form hydrogen bonds directly with the pore wall [29,51] and the mobility of these molecules may vanish due to these strong directional hydrogen bonds [26,49]. The different behaviour observed in the present simulations seems related to the fact that the considered pore lacks the ability to form hydrogen bonds directly with water molecules; this feature contributes to the increase of the number of hydrogen bonds in the contact layer and the decrease in the inner layer.

In figure 7*c*,*d*, we investigate the effect of the pore size on the dynamics of water inside the nanopores at two selected temperatures, T=310.15 K for the hydrophilic pores and T=258.15 K for the hydrophobic pores. In the case of hydrophobic confinement, we see that the axial diffusion of water molecules is faster for the smaller nanopore. On the other hand, for hydrophilic confinement, the size dependence is less pronounced. In all cases, the MSD_z_ converges to the same value for all radii of the nanopores, which is due to the pores having the same length. We conclude that the longitudinal motion of water molecules inside the pore is enhanced by confinement, i.e. for the smaller radii of hydrophobic nanopores; this seems coherent with the findings of Falk *et al*. [6] which reported that the superlubric behaviour of carbon nanotubes increases with decreasing radii.

Concerning motion in the section of the pore, the situation is reversed. The radial displacement of water molecules is now hindered by the pore wall. For both the pore sizes we thus observe that ${\text{MSD}}_{xy}$ plateaus at values that depend on the pore radius, corresponding to linear distances of *ca* 5 Å and 8.5 Å for the small and larger pore, respectively. The radial diffusion is therefore enhanced at larger radii. The total suppression of radial displacements in cylindrical nanopores can be observed in case of even stronger nanoconfinement, such as in the case of narrow carbon nanotubes that accommodate only a single-file arrangement of water molecules; there, diffusivity typically becomes negligible in the very-long time (nanoseconds) compared to axial diffusion [32,52]. Nonetheless for hydrophilic silica pores (allowing for the formation of HBs between water and the pore walls), the axial diffusion is faster in larger nanopores [29].

## 4. Conclusion

We have studied by means of molecular dynamics simulations the structure and dynamics of water confined inside model cylindrical nanopores with three levels of hydrophobicity, two different radii and at temperatures ranging from those of biological interest to colder ones.

The analysis of the structure of confined water shows that water organizes in molecular-size layers: two layers inside the pore with R=7.0 Å, and three inside the pore with R=10.2 Å. The layer close to the wall, the contact layer, shows translational order along the pore axis, induced by the pore structure. Inner layers, instead, are homogeneous along the same direction.

The spatial arrangement of water molecules in the contact layer is the most distorted as compared to bulk water due to the perturbation induced by the wall. Already from the second molecular layer away from the wall, water of inner layers recovers a bulk-like local structure despite the extreme confinement. This result supports the evidence of short-range effects (less than 5 Å) on the structure of confined water even in the absence of direct hydrogen-bond formation with the walls. A more specific analysis on the water hydrogen bonds in the smaller pore revealed a further difference between the inner and the contact layers: while molecules of the inner layers equally form hydrogen bonds with other molecules inside the same layer and in the contact layer, most of the hydrogen bonds of the contact layers are formed among molecules of this layer, which indicates that an extended hydrogen bond network is formed at the internal surface of the pore. Surprisingly, the structural properties here investigated do not change sensitively within the spanned degrees of hydrophobicity of the pore, which goes from hydrophobic to mildly hydrophilic, and in the explored range of temperatures.

On the contrary, the dynamics of confined water appears to be dependent on the hydrophobicity of the nanopore, on the radius of the nanopore, and on the temperature. Water translational dynamics along the pore axis is always faster in the smaller nanopore as compared to the larger nanopore. Radial displacements are conversely disfavoured in the smaller nanopore. The longitudinal mobility of the molecules in the contact layers is always higher than that in any other layers at all the investigated temperatures, degrees of hydrophobicity, and sizes. The finite length of the nanopore corresponds to a finite residence time of water molecules moving inside it: during their motion, water molecules can escape the cavity region and join the bulk reservoirs. This residence time depends strongly on temperature, very weakly on the hydrophobicity, and is independent of the radius of the nanopore. Nonetheless, the finite length of the nanopore appears to play a key role in posing a restriction to the axial displacement of water molecules, in the same way the wall of the nanopore poses a geometrical restriction to the radial displacements. The motion inside the nanopore is therefore effectively confined in three- dimensional space.

In conclusion, the present work demonstrates how water confined in simple pores of nanometre size can reproduce a number of peculiar features in terms of density layering, orientations, hydrogen bonds and mobility, which bear significant similarities to experimental observations of nanoconfined water.

## Data accessibility

The data supporting this manuscript can be accessed at the following link: 10.5281/zenodo.4751937.
